# Association between cannabis use and methadone maintenance treatment outcomes: an investigation into sex differences

**DOI:** 10.1186/s13293-017-0130-1

**Published:** 2017-03-30

**Authors:** Laura Zielinski, Meha Bhatt, Nitika Sanger, Carolyn Plater, Andrew Worster, Michael Varenbut, Jeff Daiter, Guillaume Pare, David C. Marsh, Dipika Desai, James MacKillop, Meir Steiner, Stephanie McDermid Vaz, Lehana Thabane, Zainab Samaan

**Affiliations:** 1 MiNDS Neuroscience Graduate Program, McMaster University, Hamilton, ON Canada; 2 Health Research Methodology Graduate Program, McMaster University, Hamilton, ON Canada; 3 Medical Science Graduate Program, McMaster University, Hamilton, ON Canada; 4 Canadian Addiction Treatment Centres, Hamilton, ON Canada; 5 Department of Medicine, McMaster University, Hamilton, ON Canada; 6 Population Genomics Program, Chanchlani Research Centre, McMaster University, Hamilton, ON Canada; 7 Department of Clinical Epidemiology and Biostatistics, McMaster University, Hamilton, ON Canada; 8 Northern Ontario School of Medicine, Sudbury, ON Canada; 9 Department of Psychiatry and Behavioural Neurosciences, McMaster University, Hamilton, ON Canada; 10 Peter Boris Centre for Addictions Research, St. Joseph’s Healthcare Hamilton, Hamilton, ON Canada; 11 Women’s Health Concerns Clinic, St. Joseph’s Healthcare Hamilton, Hamilton, ON Canada; 12 Department of Obstetrics and Gynaecology, McMaster University, Hamilton, ON Canada; 13 Cleghorn Early Intervention Clinic, St. Joseph’s Healthcare Hamilton, Hamilton, ON Canada; 14 Biostatistics Unit, Research Institute at St Joes, St. Joseph’s Healthcare Hamilton, Hamilton, ON Canada

**Keywords:** Cannabis, Opioid, Opioid use disorder, Methadone maintenance treatment, Sex differences

## Abstract

**Background:**

Cannabis will soon become legalized in Canada, and it is currently unclear how this will impact public health. Methadone maintenance treatment (MMT) is the most common pharmacological treatment for opioid use disorder (OUD), and despite its documented effectiveness, a large number of patients respond poorly and experience relapse to illicit opioids. Some studies implicate cannabis use as a risk factor for poor MMT response. Although it is well established that substance-use behaviors differ by sex, few of these studies have considered sex as a potential moderator. The current study aims to investigate sex differences in the association between cannabis use and illicit opioid use in a cohort of MMT patients.

**Methods:**

This multicentre study recruited participants on MMT for OUD from Canadian Addiction Treatment Centre sites in Ontario, Canada. Sex differences in the association between any cannabis use and illicit opioid use were investigated using multivariable logistic regression. A secondary analysis was conducted to investigate the association with heaviness of cannabis use.

**Results:**

The study included 414 men and 363 women with OUD receiving MMT. Cannabis use was significantly associated with illicit opioid use in women only (OR = 1.82, 95% CI 1.18, 2.82, *p* = 0.007). Heaviness of cannabis use was not associated with illicit opioid use in men or women.

**Conclusions:**

This is the largest study to date examining the association between cannabis use and illicit opioid use. Cannabis use may be a sex-specific predictor of poor response to MMT, such that women are more likely to use illicit opioids if they also use cannabis during treatment. Women may show improved treatment outcomes if cannabis use is addressed during MMT.

## Background

Canada is currently developing legislation for the legalization of cannabis [1]. The rationale is that legalization would have social and economic advantages by generating revenue and deterring such crimes as illegal drug dealing [2]. Prohibition has been ineffective, with data suggesting that this policy option has created more societal costs by way of excessive incarceration, largely involving already marginalized individuals [3], and no evidence to suggest that these criminal penalties have any substantial effect on public health [4].

Colorado, USA, has recently legalized cannabis, and while it remains premature to assess the public health impact of this policy, data show that the commercialization of medical marijuana in 2009 led to a 20% increase in college age (18–25 years) monthly marijuana use and a 36% increase in adult (26+ years) monthly marijuana use in the following 3 years [5]. Legalizing cannabis will almost certainly increase its availability and accessibility; plausible mechanisms for increasing recreational use include reduced prices, ease of access, criminal penalties no longer acting as a deterrent, and increased social acceptability [6]. It is reasonable to expect that Canada will observe a similar increase in the prevalence of cannabis use, though its public health impact remains uncertain.

Despite the commonly held perception that cannabis is relatively harmless [7], its use has been linked to adverse consequences such as cognitive impairment, lower life satisfaction, respiratory problems, and increased risk of developing psychotic episodes and disorders [8]. Those with a history of psychiatric or substance-use disorders can experience worsened symptoms from cannabis use [1]. Cannabis users are also at heightened risk for developing other substance-use disorders [9]. However, the current system of criminalization is similarly associated with individual and public risks. For example, individuals with a criminal record from minor possession charges often experience considerable difficulties in finding employment or housing leading to further social and health risks [1]. Public costs of criminalization are also substantial, with an estimated $2.3 billion spent annually on enforcement and prosecution [1].

While public health risks of cannabis legalization may by and large be minimal, certain vulnerable populations are more susceptible to the deleterious effects of its use. One such population are those with substance-use disorders. North America is currently in the midst of an opioid crisis [10], in which we are witnessing a dramatic increase in non-medical use of opioids and subsequently the incidence of opioid use disorder (OUD). While opioid abuse is associated with serious adverse outcomes, it has been shown that the development of addiction is a major driver in the increase in opioid-related morbidity and mortality [11], indicating the extent to which OUD negatively impacts public health.

Because of the ongoing opioid epidemic in Canada, we must remain mindful of how increasing accessibility of cannabis will impact this population, in particular. Currently, the most commonly prescribed treatment for OUD is methadone maintenance treatment (MMT), an opioid substitution therapy [12]. MMT has proven to be effective in retaining patients in treatment and reducing opioid use and mortality [13], and this effectiveness has led to a steep increase in patients on MMT. In Ontario, Canada, the number of patients receiving MMT has nearly doubled since 2010 [12]. Despite its effectiveness, a significant number of patients respond poorly to treatment and experience relapse [14]. Illicit opioid use in combination with MMT is of immense concern, as it is a substantial risk factor for overdose and death [15].

Recent studies point to a changing landscape of OUD and those in treatment, one that includes a higher percentage of women, older aged patients, and more individuals abusing prescription opioids rather than heroin [16]. These sociodemographic changes warrant a re-evaluation of risk factors associated with poor MMT outcomes.

Compared to the general population, patients on MMT show a higher prevalence of cannabis use [16], and because of its documented association with polysubstance use [9, 17], psychiatric disorders [18], and overall worse quality of life [19], represents a potential risk factor for poor MMT outcomes. Several studies have investigated the influence of cannabis use on MMT outcomes in humans, though the results are mixed. Some studies have indicated cannabis use is associated with poorer treatment outcomes [20–22] while others looking at illicit opioid use found no significant association [23–26]. Although this is the case, confidence in these diverging results is reduced by methodological limitations such as small sample size and subjective outcome measures, making further investigations merited.

Furthermore, few studies have considered sex as a potential moderator. It is well established that substance-use behaviors differ by sex and different social and biological factors contribute to the development of substance-use disorders between men and women [27]. Although a higher proportion of men use cannabis, women who use cannabis are more likely to experience adverse outcomes such as development of cannabis use disorder, and may also be more likely to show negative outcomes from cannabis in other domains such as more severe cannabis withdrawal symptoms and [28] and worse mental health and social functioning [29]. A large survey of cannabis users, for example, found that a larger proportion of men use cannabis for recreational purposes while more women reported using it for purposes of self-medication [30]. Thus, motivational processes for drug use may differ between men and women.

The objective for this study is to investigate sex differences in the association between cannabis use and illicit opioid use during methadone maintenance treatment. We will build on previous research by including a large, representative sample of MMT patients to ensure adequate power and generalizability of findings. Our secondary objective is to determine whether heaviness of cannabis use is associated with illicit opioid use among male and female cannabis users.

## Methods

### Participants and procedure

**Fig. 1:**
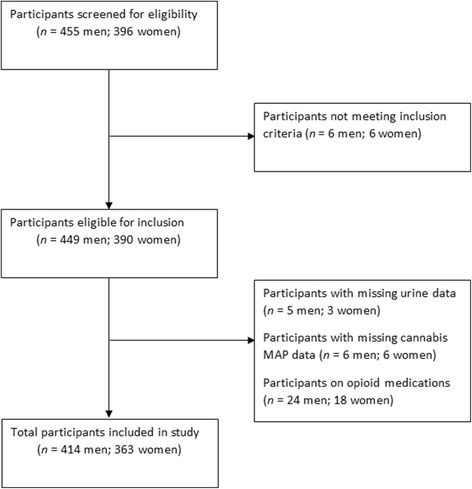
Flow diagram for eligibility and screening of participants

Data were collected as part of the Genetics of Opioid Addiction (GENOA) program, an ongoing prospective cohort study conducted in collaboration with the Population Genomics Program at McMaster University, and the Canadian Addiction Treatment Centre (CATC) [31]. We recruited participants from 16 CATC sites across Ontario, Canada, from 2013 to 2016. Patients were eligible for participation if they were ≥18 years old, on methadone maintenance treatment for OUD, and able to provide informed written consent. Individuals were excluded if they did not speak English, were on an opioid substitution therapy other than methadone, or refused to provide blood or urine samples (Fig. 1). If individuals were deemed eligible for participation, they were provided with a written consent form to read and sign. Eligible participants provided informed written consent, upon which they underwent a face-to-face interview administered by trained research staff. Participants were compensated with a 5$ coffee shop gift card. This study was approved by the Hamilton Integrated Research Ethics Board (HIREB; Study ID 11-056).

### Data collection

The study participants provided sociodemographic and clinical information during the face-to-face interview. Participants were asked to report their biological sex, and all participants reported either male or female. We also collected information regarding current methadone maintenance treatment, methadone dose, duration of current treatment, and information about any past treatments for opioid use disorder.

The Maudsley Addiction Profile (MAP) [32] was administered to retrieve information about substance-use, health risk behaviors, physical and psychological health, and personal and social functioning in the past 30 days. Substance-use data included information on number of days used in the past 30, typical dose used, and route of administration. We also used the physical and psychological health sections of the MAP to compare general health and well-being among participants. These sections comprised of eight questions each and were scored using a Likert scale ranging from 0 to 4 (never-always) to produce a maximum score of 40 per section.

All study data were collected and managed by trained researchers using REDCap electronic data capture tools [33].

### Drug use measurements

In addition to self-reported use of drugs using the MAP, all study participants underwent routine weekly or biweekly urine toxicology screens at the clinical sites part of routine clinical care as per CATC management protocol.

#### Cannabis use

Cannabis use, the primary predictor variable, was measured using urinalysis (cut-off = 50 ng/ml for tetrahydrocannabinol) in the past 3 months. Unfortunately, several clinics discontinued screening for cannabis during urine testing, so only 45.0% of participants had any cannabis urine screens. Therefore, we opted to use self-reported cannabis use from the MAP. To verify the validity of self-reports, we calculated the sensitivity and specificity using participants who had data for both urinalysis and MAP (*n* = 349). The sensitivity was 79.9% (95% CI 72.7, 85.8) and specificity was 80.0% (95% CI 73.6, 85.4), and thus we deemed self-reported cannabis use an appropriate measure of cannabis use. Sensitivity and specificity values did not significantly differ between men and women, and there were no significant differences between false negatives and false positives.

For the primary regression analysis, we dichotomized cannabis use as any reported use versus no use in the past 30 days for our main predictor variable. We defined heaviness of cannabis use as the product of number of days used in the past 30 days by the typical dose per use (measured in grams) as reported on the MAP.

To quantify cannabis heaviness for participants who reported doses in values other than grams, we utilized the quantification of common “marijuana measurements” as determined and reported by Mariani et al. [34]. Many participants reported values such as “less than one joint” or “couple of puffs of a joint”, and we coded all of these reports as equivalent to one half of a joint (0.33 g). For all other reported quantities, we consulted an addiction expert to estimate the average dose per route of administration based on clinical experience. We used the following quantifications: bowl = 0.25 g and cookie = 2 g.

#### Illicit opioid use

Illicit opioid use during MMT was the primary outcome which was measured in the 3 months prior to baseline interview using urinalysis, with participants averaging 16 screens per 3 months. The cut-off concentration was 300 ng/mL for opiates and 100 ng/mL for oxycodone. We dichotomized illicit opioid use to reflect no positive screens versus any positive screens during a 3-month duration. This dichotomized variable is a patient-important treatment outcome, as the ultimate goal of MMT is complete abstinence of opioids. Individuals were excluded from analysis if they were currently prescribed any opioid medications, as these compromise the results of urine screens.

### Statistical analysis

Descriptive statistics were reported to compare demographic characteristics between men and women. Continuous variables were expressed as mean (standard deviation) and categorical variables were expressed as number (percent). We employed a Student’s *t* test to test significant differences between continuous variables, and a chi-square test for categorical variables.

A multivariable logistic regression analysis was performed to investigate the association between cannabis and illicit opioid use, including an interaction term, sex by cannabis use, to investigate between-group sex differences. In the analysis, we controlled for age, sex, methadone dose, and treatment duration. Two multivariable logistic regression analyses were also performed for men and women separately to investigate within-group sex differences, controlling for the same covariates.

We conducted a secondary analysis on cannabis users to determine whether it is only the presence of cannabis use that influences treatment outcome or the heaviness of use that drives the association. For this, we replaced the binary cannabis variable with the continuous measurement of cannabis use heaviness. Multivariable logistic regression analyses were employed for male and female users, controlling for the same covariates as in the initial analysis.

Variables were assessed for collinearity using the variance inflation factor (VIF), and variables with VIF > 10 were excluded from the analysis. Adjusted odds ratios (OR), 95% confidence intervals (CI), and *p* values generated from the regression models are reported. The level of significance for hypothesis testing was set at alpha = 0.05 for the main analysis and alpha = 0.025 for analyses performed separately on men and women.

The general requirement for logistic regression is to have a minimum of 10 events per predictor variable [35]. We included 212 men and 183 women with the event (presence of at least one positive opioid urine screen), and we included four predictor variables therefore the study was adequately powered for analysis. When isolating cannabis users for the secondary analysis, there were 133 men and 91 women with the event, demonstrating adequate power.

All analyses were performed using IBM SPSS version 20. This study is reported in adherence to the Strengthening the Reporting of Observational Studies in Epidemiology (STROBE) guidelines [36].

## Results

### Participants’ characteristics

The total sample comprised of 777 participants including 414 men and 363 women (Fig. 1). Ages varied from 18 to 65 years with a mean age of 38.05 years (SD =11.11). The mean daily methadone dose was 75.44 mg (SD = 45.84), and the average duration of current MMT was 48.55 months (SD = 49.53).

Demographic and clinical characteristics comparing men and women are reported in Table 1. 59.7 of males and 43.5% of females reported using cannabis. Furthermore, men on average used cannabis more often in the past 30 days and at a higher average dose. Women also had significantly worse physical and psychological functioning compared to men. A comparison of cannabis users and non-users can be found in Appendix 1.

**Table 1:** Demographic and clinical characteristics of men and women on MMT

Variable	Men (*n* = 414)	Women (*n* = 363)	*p* value
Age in years (SD)	39.07 (11.72)	36.88 (10.27)	0.006
Ethnicity (% Caucasian)	347 (84.6%)	288 (80.2%)	0.127
Marital status			
Never married (%)	203 (49.0%)	158 (43.5%)	0.079
Married/common law/living with partner (%)	129 (31.2%)	109 (30.0%)	
Widowed/separated/divorced (%)	82 (19.8%)	96 (26.4%)	
Education			
Less than grade 9 (%)	88 (21.4%)	68 (18.9%)	0.008
Grade 9–12 (%)	233 (56.6%)	177 (49.2%)	
Trade school, college, university (%)	91 (22.1%)	115 (31.9%)	
Employment (% currently working)	175 (42.3%)	98 (27.0%)	<0.001
Smoking status (% current smoker)	336 (81.2%)	320 (88.2%)	0.007
Age of onset of opioid use in years (SD)	24.90 (8.90)	25.00 (8.11)	0.881
Methadone dose in mg/day (SD)	78.15 (48.36)	72.34 (42.63)	0.079
Current treatment duration in years (SD)	4.10 (4.11)	3.98 (4.15)	0.704
Physical functioning (SD)	14.45 (7.74)	16.79 (7.38)	<0.001
Psychological functioning (SD)	12.33 (8.82)	15.11 (9.36)	<0.001
Cannabis use (% cannabis users)	247 (59.7%)	158 (43.5%)	<0.001
Days cannabis use in last 30 (SD)	11.97 (13.54)	7.44 (12.02)	<0.001
Average cannabis dose in g/day (SD)	1.48 (1.71)	1.04 (1.03)	0.004

Maximum score for the MAP physical and psychological functioning is 40, with higher scores indicating worse functioning

*SD* standard deviation

### Cannabis use

The primary logistic regression analysis did not yield a significant association between cannabis use and illicit opioid use, after adjusting for age, sex, methadone dose, and treatment duration (OR = 1.16, 95% CI 0.77, 1.75, *p* = 0.49). The interaction of sex and cannabis use also did not show a significant association with illicit opioid use in the regression model (OR = 1.52, 95% CI 0.84, 2.77, *p* = 0.17) (Table 2).

**Table 2:** Multivariable logistic regression analysis on predictors of illicit opioid use

Predictor	Odds ratio	95% CI	*p* value
Cannabis use	1.16	0.77–1.75	0.485
Sex*cannabis use	1.52	0.84–2.77	0.169
Age	1.00	0.99–1.02	0.857
Sex	0.83	0.54–1.28	0.399
Methadone dose	0.96*	0.93–0.99	0.023
Duration of treatment	0.91*	0.87–0.95	<0.001

Age and duration of treatment interpreted as a one-point increase. Methadone dose interpreted as a 10-point increase

*Significant at *p* < 0.05

*OR* odds ratio, *CI* confidence interval

### Sex differences

After adjusting for age, methadone dose, and treatment duration, any cannabis use in the past 30 days was significantly associated with illicit opioid use (OR = 1.82, 95% CI 1.18, 2.82, *p* = 0.007) in women but not in men (OR = 1.11, 95% CI 0.73, 1.69, *p* = 0.62) (Table 3).

**Table 3:** Multivariable logistic regression analysis on predictors of illicit opioid use by sex

	Men			Women		
Predictor	Odds ratio	95% CI	*p* value	Odds ratio	95% CI	*p* value
Cannabis use	1.11	0.73–1.69	0.618	1.82*	1.18–2.82	0.007
Age	0.99	0.98–1.01	0.588	1.01	0.99–1.03	0.356
Methadone dose	0.94*	0.90–0.99	0.010	0.99	0.94–1.04	0.634
Duration of treatment	0.92*	0.87–0.97	0.004	0.90*	0. 84–0.95	<0.001

Age and duration of treatment interpreted as a one-point increase. Methadone dose interpreted as a ten-point increase

*OR* odds ratio, *CI* confidence interval

*Significant at *p* < 0.025

### Heaviness of cannabis use

Among cannabis users, the mean number of days of cannabis use in the past 30 days was 18.91 days (SD = 12.46) and the mean daily dose was 1.31 g (SD = 1.50), varying from 0.10 to 14.00 g. The logistic regression analysis showed the heaviness of cannabis use to be unrelated to illicit opioid use in both women (OR = 1.00, 95% CI 0.99, 1.01, *p* = 0.92) and men (OR = 1.01, 95% CI 1.00–1.01, *p* = 0.07) (Table 4).

**Table 4:** Multivariable logistic regression analysis on predictors of illicit opioid use among cannabis users by sex

	Men			Women		
Predictor	Odds ratio	95% CI	*p* value	Odds ratio	95% CI	*p* value
Cannabis use heaviness	1.01	1.00–1.01	0.072	1.00	0.99–1.01	0.917
Age	0.99	0.97–1.02	0.476	1.02	0.98–1.05	0.449
Methadone dose	0.92*	0.87–0.98	0.016	1.02	0.94–1.11	0.662
Duration of treatment	0.91	0.84–0.99	0.037	0.91	0.83–0.99	0.035

Cannabis use heaviness, age, and duration of treatment interpreted as a one-point increase. Methadone dose interpreted as a 10-point increase

*Significant at *p* < 0.025

## Discussion

The current study sought to investigate sex differences in the association between cannabis use and illicit opioid use in a cohort of MMT patients. Our results suggest that cannabis use during treatment may be a predictor of illicit opioid use in women. This could help explain why previous studies investigating this relationship provided conflicting results due to the lack of consideration of sex effect on the association between cannabis use and continued opioid use in MMT [23, 37].

To our knowledge, this is the largest study conducted to date investigating the relationship between cannabis use and illicit opioid use in men and women on MMT. While some studies have indicated that cannabis use is associated with poor MMT treatment outcomes [20–22], several previous studies looking at illicit opioid use have not found significant results [23, 24, 26]. These inconsistent reports could be explained by methodological limitations such as the selection of the study participants [23] and insufficient investigations into sex differences in cannabis use and MMT treatment outcomes. For example, the external validity of the studies reporting no association may be low, as two were secondary analyses of RCTs with restrictive inclusion criteria [23, 26], and one study analyzed a sample of predominantly men [24]. In this case, it is unlikely these findings apply to a current sample of MMT patients which contain about 50% women.

Despite the well-documented sex differences in the sociodemographic and clinical profiles of patients in MMT [38], there has been little research conducted on sex-specific predictors of MMT outcomes. Women are more sensitive to the subjective effects of cannabis (i.e., subjective ratings of intoxication and other drug effects like altered mood and sociability) and consequently show a faster trajectory to cannabis use disorder [28], indicating they may be have a higher proclivity to problematic cannabis use. Furthermore, cannabis use has consistently been shown to be associated with worse mental health outcomes in women compared to men [19, 39].

Preclinical research points to many important developmental and biological sex differences which suggest females are more susceptible to the deleterious effects of cannabis use. Studies in rodents have found that females exposed to ∆9-tetrahydrocannabinol (THC) were more susceptible to the reinforcing effects of cannabinoids, such that female rats more quickly acquired self-administration and were more sensitive to drug- and cue-induced reinstatement of the drug [40]. These behavioral observations may be explained by the findings that prolonged exposure to THC led to a much greater cannabinoid receptor desensitization in female rats compared to their male counterparts [40]. It was also found significantly greater concentrations of THC and its metabolites in the female rat brain compared to males [41]. Despite this evidence, there is a paucity of research looking into the sexually dimorphic effects of cannabis in humans [42].

While there is reason to consider biological mechanisms as explanation for the differential consequences of cannabis use in men and women, other clinical and social factors should not be overlooked. Women in MMT tend to show a higher prevalence of comorbid psychiatric and physical illnesses [16, 43, 44], as well as more severe opioid craving upon treatment entry [45] which may represent confounding factors that serve to increase rates of both cannabis and opioid use during MMT. As such, these patients may have motivation to use both drugs for purposes of self-medication. Indeed a survey of cannabis users found men were more likely to use cannabis recreationally while women were more likely to use it for purposes of self-medication for conditions such as anxiety and headaches [30]. As we only classified participants based on biological sex, further work should evaluate gender constructs and their influence on treatment response to determine whether the observed sex differences can be explained by biological or social mechanisms, or a combination of the two.

Unexpectedly, when looking at cannabis users only, we failed to find an association between heaviness of cannabis use and illicit opioid use in either sex. It is currently unclear why this is the case. A study by Saxon et al. [46] found that MMT patients who had intermittent positive cannabis urine screens had a significantly higher percentage of positive screens for other drugs of abuse compared to those who consistently had positive screens. Thus, the relationship between cannabis use heaviness and illicit opioid use may not be linear. On the other hand, this observation may simply be the result of our rough approximation of cannabis use heaviness and slang terminology reported in the interviews, rather than reflecting the true effect.

Several studies also indicate a distinct difference between recreational cannabis users and those with cannabis use disorder, regardless of frequency of use, such that patients with a cannabis use disorder actually show less polysubstance use during MMT [23, 47, 48]. It is unclear why this is the case, but it may represent a confounding effect such as having cannabis use disorder may be associated with lack of means to obtain further drugs and lack of will or time to use other drugs while on MMT. In this study, we did not find a significant association between the amount or frequency of cannabis use and illicit opioid use. However, our study lacks the ability to distinguish cannabis use disorder from recreational use.

Another consideration is to account for the potency of cannabis used by patients, which was not measured in this study. Research on opioid-dependent rats suggests cannabidiol (CBD) and THC, the two main active ingredients in cannabis, actually generate opposing response. Administration of CBD extinguishes cue-induced heroin-seeking behaviors following periods of abstinence [49], whereas THC administration seems to heighten opioid sensitivity and increase heroin self-administration [50, 51]. This antagonism is further supported by imaging studies in humans, which suggest that CBD attenuates the neurotoxic and adverse psychiatric effects of THC [52, 53]. Because of these differential effects, those who use cannabis for medicinal purposes may choose higher CBD concentrations while those who use it for recreational purposes may prefer greater amounts of THC. Therefore, depending on ratio of CBD to THC in the ingested cannabis, an individual may become more or less susceptible to further drug use, and this distinction should be investigated further.

Some limitations of this study should be noted. The cross-sectional nature of the analysis prevents any causal inferences from being made. Self-reported cannabis use, despite its adequate sensitivity and specificity may also be a biased estimate. Particularly in chronic cannabis users, short-term memory and recall may be impaired [54, 55] which could affect the accuracy of retrospective self-reports even further. Conversely, there is evidence to suggest self-report use may be a more valid and sensitive indicator of cannabis use compared to urine screening. For example, patients enrolled in methadone maintenance treatment are required to provide urine samples at least one or two times per week; however, studies have shown the average time for the first negative result in urine screening for THC metabolites following a single dose of THC was 8.5 days following ingestion for infrequent users and 19.1 days for chronic users [56]. This suggests that urine data may overestimate the frequency of cannabis use.

## Conclusions

This study suggests that cannabis use is a potential sex-specific predictor of poor outcome during MMT. It will be important to look at the impact of cannabis use on women by systematically screening for cannabis use in women with OUD and providing addiction counseling to address not only opioid use but also cannabis use in this vulnerable group. This study also showed that women with OUD experienced physical and psychological symptoms more frequently than men; these symptoms may be the underlying cause of cannabis use in women in this study and addiction services should consider sex-specific treatment programs to manage symptoms and co-substance use.

## Appendix 1

**Table 5:** Demographic and clinical characteristics of cannabis users and non-users on MMT

Variable	Cannabis non-users (*n* = 372)	Cannabis users (*n* = 405)	*p* value
Age in years (SD)	39.78 (11.05)	36.46 (10.94)	<0.001
Sex (% female)	205 (55.1%)	158 (39.0%)	<0.001
Ethnicity (% Caucasian)	306 (83.4%)	329 (81.8%)	0.634
Marital status			
Never married (%)	150 (40.3%)	211 (52.1%)	0.004
Married/common law/living with partner (%)	126 (33.9%)	112 (27.7%)	
Widowed/separated/divorced (%)	96 (25.8%)	82 (20.2%)	
Education			
Less than grade 9 (%)	67 (18.2%)	89 (22.0%)	0.087
Grade 9–12 (%)	190 (51.6%)	220 (54.5%)	
Trade school, college, university (%)	111 (30.2%)	95 (23.5%)	
Employment (% currently working)	132 (35.5%)	141 (34.8%)	0.880
Smoking status (% current smoker)	301 (80.9%)	355 (87.7%)	0.010
Age of onset of opioid use in years (SD)	26.12 (9.08)	23.86 (7.86)	<0.001
Methadone dose in mg per day (SD)	78.77 (46.54)	72.36 (45.02)	0.053
Current treatment duration in years (SD)	4.26 (4.35)	3.85 (3.91)	0.164
Physical functioning (SD)	15.06 (7.92)	16.02 (7.38)	0.085
Psychological functioning (SD)	12.90 (9.57)	14.27 (8.76)	0.040

Maximum score for the MAP physical and psychological functioning is 40, with higher scores indicating worse functioning

*SD* standard deviation

## Abbreviations

CATC: Canadian Addiction Treatment Centre
CBD: Cannabidiol
CI: Confidence interval
GENOA: Genetics of Opioid Addiction
HIREB: Hamilton Integrated Research Ethics Board
MAP: Maudsley Addiction Profile
MMT: Methadone maintenance treatment
OR: Odds ratio
OUD: Opioid use disorder
SD: Standard deviation
STROBE: Strengthening the reporting of observational studies in epidemiology
THC: ∆9-tetrahydrocannabinol
VIF: Variance inflation factor
